# Presymptomatic type 1 diabetes and disease severity at onset

**DOI:** 10.1007/s00125-023-05999-0

**Published:** 2023-09-26

**Authors:** Josephine Schneider, Gita Gemulla, Wieland Kiess, Reinhard Berner, Angela Hommel

**Affiliations:** 1 Department of Pediatrics, Faculty of Medicine and University Hospital Carl Gustav Carus, Technische Universität Dresden, Dresden, Germany; 2 Center for Regenerative Therapies Dresden, Faculty of Medicine, Technische Universität Dresden, Dresden, Germany; 3 Hospital for Children and Adolescents, University of Leipzig, Leipzig, Germany; 4 Center for Pediatric Research, University of Leipzig, Leipzig, Germany

**Keywords:** Diabetic ketoacidosis, Islet autoantibodies, Type 1 diabetes

*To the Editor:* The recent findings of Hummel et al demonstrate the potential clinical benefits of identifying children with presymptomatic type 1 diabetes [1]. The authors compared children who developed type 1 diabetes after a previous diagnosis of presymptomatic type 1 diabetes in the Fr1da study with those in a registry cohort of children diagnosed without a prediagnosis. Assessment of the advantages and disadvantages of screening should consider both benefit to the patient and to the healthcare system. Important patient-relevant clinical endpoints include diabetic ketoacidosis (DKA), symptoms and hospitalisation, none of which were reported in the comparative population of children without a previous diagnosis of presymptomatic type 1 diabetes [1].

Since 1999, the Childhood Diabetes Registry of Saxony, Germany has collected information on 2890 newly diagnosed cases of diabetes in children and adolescents aged ≤17.99 years, with 98% ascertainment between 2013 and 2019 [2]. Here, we examined the data from individuals entered into this registry who were diagnosed with recent-onset type 1 diabetes aged ≤10.99 years, to provide comparative information from a neighbour region to Bavaria (where the Fr1da study was set) with respect to DKA, clinical symptoms and hospitalisation (Table 1). A total of 907 children diagnosed with type 1 diabetes were registered between June 2013 and June 2023. Data for DKA and hospitalisation were entered for 885 children, whilst data for symptoms were entered for 904 children. DKA, indicated by a blood pH <7.3, was observed in 333 (37.6%) of the 885 children, including 116 (13.1%) with severe DKA (blood pH <7.1). Symptoms were reported in 865 of 904 (95.7%) children and all 885 children with hospitalisation data were found to be hospitalised for an overall median of 11 days (IQR: 9–14 days). In comparison, in the Fr1da study, in children with a diagnosis of presymptomatic type 1 diabetes, 2.5% presented with DKA (*p*<0.0001 vs Childhood Diabetes Registry of Saxony, analysed by *χ*^2^ test using MedCalc, Version 22.009 [3]), 43.8% presented with symptoms (*p*<0.0001) and the median time of hospitalisation was 8 days [1]. A similar islet autoantibody screening approach has recently been introduced in Saxony as was used in the Fr1da study. Since 2020, six children developed type 1 diabetes between the ages of 2 and 4 years after a previous diagnosis of presymptomatic type 1 diabetes, which represents 5% of all type 1 diabetes diagnoses below 5 years of age in the Childhood Diabetes Registry of Saxony. Consistent with the data from the Fr1da study, which showed that very few children (2.5%) presented with DKA at diagnosis [1], none of these six children had DKA at presentation. These data add to the evidence [1, 4] that widespread adoption of sensitive and specific screening for islet autoantibodies, and follow up of children with multiple islet autoantibody-positive presymptomatic type 1 diabetes have the potential to markedly reduce disease severity at clinical onset.

**Table 1:** Age and clinical characteristics at diagnosis in children diagnosed with type 1 diabetes before 11.0 years of age

Characteristic	*n* with data	*n* (%) or median (IQR)
Age at diagnosis (years)	907	6.9 (4.1–9.1)
DKA	885	
No (pH ≥7.3)		552 (62.4)
Yes (pH <7.3)		333 (37.6)
Severe (pH <7.1)		116 (13.1)
Hospitalisation (days)	885	11 (9–14)
Symptoms	904	
No		39 (4.3)
Yes		865 (95.7)

Data from the Childhood Diabetes Registry of Saxony

## Data Availability

The data that support the findings presented in this letter are not openly available due to reasons of sensitivity and are available from the corresponding author upon reasonable request.

## Abbreviation

DKA: Diabetic ketoacidosis
